# The occurrence of pharmacologically active dyes in the aquatic environment as a result of water and wastewater contamination—an underestimated environmental problem

**DOI:** 10.1007/s11356-026-37866-x

**Published:** 2026-06-12

**Authors:** Angelika Tkaczyk-Wlizło, Kamila Mitrowska

**Affiliations:** 1 Department of Chemical Research of Food and Feed, National Veterinary Research Institute (PIWet), Al. Partyzantów 57, Puławy, Poland; 2 Institute of Biological Bases of Animal Production, University of Life Sciences in Lublin, Akademicka 13, 20-950 Lublin, Poland

**Keywords:** PAD, PAS, Aquatic contamination, Toxicity, Food web

## Abstract

Due to colouring properties, synthetic organic dyes are omnipresent in many application areas, from the textile, tannery, and cosmetic to the food industries. Among them, some compounds are pharmacologically active dyes (PADs) used against selected pathogenic bacteria, fungi, and parasites. However, some of the PADs also have toxicological properties; therefore, their usage in human and veterinary medicine is restricted. The large-scale production of PADs and widespread applications have caused these dyes to permeate the aquatic environment. Despite few reports confirming PADs occurrence in bottom sediment, wild fish, and water, they are still underestimated as emerging contaminants of the water bodies. Moreover, PADs as synthetic organic dyes are stable to light and resistant to microbial treatment; hence, their decolourisation and degradation are difficult in wastewater treatment plants (WWTPs). Up to now, PADs have been found in various types of municipal and industrial wastewater. Due to the pharmacological activity and toxicological properties of some PADs and their confirmed occurrence in the aquatic environment, their presence in the environment should be monitored. The hazard potential of PADs should be assessed, considering individual levels of the aquatic food web with particular regard to tertiary consumers such as predatory fish and humans as top-level consumers. This review collects scientific data considering applications of PADs in human and veterinary medicine and their occurrence in different parts of the aquatic environment, aquaculture, and wastewater. Also, the toxicity of PADs on the individual level of the aquatic food web and risk assessments of selected dyes are presented.

## Introduction

**Fig. 1:**
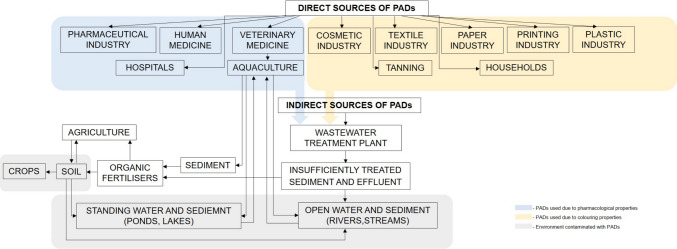
Direct and indirect sources of PADs in the aquatic environment

Among colouring substances, synthetic organic dyes are the largest group, with global production exceeding 1,000,000 tons annually. They are widely used in the textile, tanning, plastic, printing, and paper industries; some also have found applications in cosmetics and food processing (Dutta et al. 2022). Moreover, some synthetic organic dyes are also pharmacologically active substances (PASs) and are applied in human and veterinary medicine (Fig. 1) (Tkaczyk et al. 2020; Wainwright 2021).

The application of pharmacologically active dyes (PADs) as therapeutic compounds has a long history. The acridine dye, acriflavine, was reported as a trypanocide in 1912 by Ehrlich and Benda. After that, acriflavine and proflavine were introduced as wound antiseptics in hospitals during World War One (Browning et al. 1922; Wainwright 2001). Recently, acriflavine has been reported as an inhibitor of the virus SARS-CoV-2 (Napolitano et al. 2022). Balabanova et al. (2003) reviewed applications of PADs in human dermatology; dyes such as brilliant green, fuchsin, malachite green, and toluidine blue indicate topical antimicrobial properties. Methylene blue, one of the most well-known phenothiazine dyes, was used in several important clinical areas, including therapeutics for Alzheimer’s disease, malaria and schizophrenia. Currently, methylene blue poses an important photosensitiser in the photodynamic therapy of cancer and, more recently, of photodynamic antimicrobial chemotherapy (Schirmer et al. 2011; Wainwright 2005).

The most recognisable PAD in veterinary medicine is a triphenylmethane dye—malachite green. In 1933, Foster and Woodbury (1936) reported that using malachite green was very effective against fungus infections in fish and fish eggs. At the beginning of 1980, Alderman tested different PADs such as brilliant green, crystal violet, ethyl violet, malachite green, rhodamine B, Victoria blue 4R, and thioflavine impact on fish parasitic fungi activity. Among the abovementioned PADs, triphenylmethane dyes were the most effective (Alderman 1982). Due to activity against eggs and fish mould infections, PADs such as acriflavine, malachite green, and methylene blue have also been used as disinfectants in ornamental fish disease treatment (Noga 2010).

It is worth emphasising that any dyes have never been registered as veterinary medicinal products for food-producing animals due to their toxicological potential (European Commission 2010; IARC 2010; 2016; Le Curieux et al. 2021; Meyer And Jorgenson 1983; NTP 2004). Due to their toxic properties, PADs should be used only in animals not intended for human consumption. Unfortunately, the high effectiveness, easy availability, and low cost of the dyes caused their continued illegal use in fish (Bojarski et al. 2023; Dubreil et al. 2019; Fallah And Barani 2014; Kwan et al. 2018), frogs (Turnipseed et al. 2012), sea snails (Fei et al. 2022), shrimp (Uddin And Kader 2006), and turtles farming (He And Cui 2016; Shen et al. 2019).

Most of the PADs, despite their pharmacological activity, have colouring properties. Due to that, they are used on a large scale in different sectors such as the textile, tannery, cosmetic, plastic, printing, and paper industries. After applications of dyes, their residues are present in industrial wastewater (Hakami et al. 2020; Khan et al. 2019; Razmara et al. 2011). Some PADs, such as auramine-O and malachite green, are used as germicides to clean courtyards, houses, and temple premises in India (Hisham et al. 2018; Mohideen et al. 2015). Therefore, inappropriate usage of PADs as therapeutic agents or to their colour to dye clothes, hair, etc., caused PADs to be also found in municipal wastewater (Chiang et al. 2011; Razmara et al. 2011). PADs, as synthetic organic dyes, have been designed to resist various factors, such as light and microbial attacks. Thus, dyeing is permanent on items, e.g. clothes. As a result, dye degradation during different wastewater treatment plant (WWTP) processes is complex (Shaffiqu et al. 2002).

All mentioned factors caused dyes to permeate to different compartments of the aquatic environment. Up to now, PADs have been found in environmental water such as rivers (Azni et al. 2024; Bagtash And Zolgharnein 2018; Chiang et al. 2011), lakes (Hakami et al. 2021; Tkaczyk-Wlizlo And Mitrowska 2023), and seawater (Hashemi et al. 2019; Seraj et al. 2022) and fish farming water (Es'haghi et al. 2011; Hakami et al. 2021; Yu et al. 2015).

The toxicological studies showed that among different PADs, malachite green is the most dangerous for aquatic organisms on varying levels of the food web. The occurrence of dyes in the aquatic environment is not neutral for living organisms, including humans (Hashimoto et al. 2011; Srivastava et al. 2004b). The pharmacological activity and toxicological properties of some of PADs which caused their presence in the aquatic environment should be monitored. There is a need to include PADs in environmental legislation, and their occurrence in WWTPs should be controlled (Tkaczyk et al. 2020).

Therefore, the aim of this study is to emphasise an environmental problem resulting from the use of PADs with particular regard to their occurrence in aquatic environment, aquaculture, and wastewater. Also, the toxicity of PADs on the individual level of the aquatic food web and risk assessments of selected dyes are presented.

## PADs classification and basic pharmaceutical applications

Among different chemical classes of synthetic organic dyes, only five of them: azo, acridine, phenothiazine, triphenylmethane, and xanthene are considered PADs (EFSA et al. 2017, Mezgebe And Mulugeta 2022; Verdon And Andersen 2017). The dye examples of the mentioned chemical classes and their basic properties are presented in Table 1.

**Table 1:**
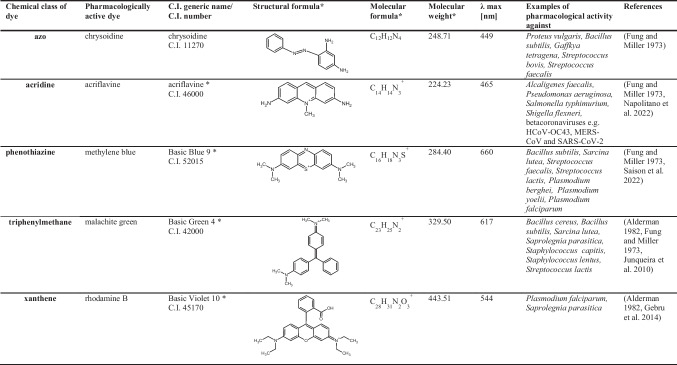
Chemical classes of PADs with their examples

*Refers to the cationic form of a pharmacologically active dye

The vast majority of PADs, such as acriflavine, crystal violet, malachite green, and methylene blue, are cationic dyes and have activity against bacteria (Fung And Miller 1973; Junqueira et al. 2010), fungi (Alderman 1982), parasites (Gebru et al. 2014; Saison et al. 2022), and viruses (Napolitano et al. 2022), and act as antiseptics (Balabanova et al. 2003); hence, they are applied in human and veterinary medicine (Table 1) (Verdon And Andersen 2017; Wainwright 2021). The dyes’ action mechanism is based on the electrophilic centre of the molecule, which forms adducts with nucleophilic macromolecules such as DNA. The biocidal effect of PADs resulted from the inhibition of respiratory enzymes of disease-causing organisms (Del Castillo et al. 2010; Mitrowska And Posyniak 2005).

In human medicine, phenothiazine dyes, especially methylene blue, are used as photosensitisers in photodynamic therapy of cancers and photodynamic antimicrobial chemotherapy. The photosensitiser dyes are also used in the donated blood sample as a method of eradication from antimicrobial infections (Wainwright 2008). The antiseptic properties of acridine dyes (acriflavine, proflavine) have been well known since World War One. Recently, acriflavine has also been successfully tested against the virus SARS-CoV-2 (Napolitano et al. 2022).

On the other hand, the conducted studies in veterinary medicine have shown that among different available PADs, malachite green is the most effective dye against fungi infections, especially *Saprolegnia parasitica* in fish and fish eggs (Alderman 1982; Noga 2010). Also, this dye shows antimicrobial activity in shell disease of lobsters (Kim et al. 2018). Malachite green and other PADs, however, can potentially be harmful to humans. Hence, in many countries, including the European Union, applying PADs in veterinary medicine is limited only to fish not intended for human consumption (European Commission 2010, Meyer And Jorgenson 1983, NTP 2004). Another triphenylmethane dye, crystal violet, has been used in poultry farming for years to prevent poultry feed moulding (Roybal et al. 1989). However, when the poultry is farmed for human consumption, the use of the dye is illegal.

## Illegal PADs in aquaculture

The intensive aquaculture production caused the application of pharmaceuticals to be unavoidable. PADs such as malachite green, crystal violet, brilliant green, or methylene blue are effective and of low cost; hence, they are illegally used to prevent and treat microbial infections (Dutta And Bhattacharya 2021; Rasul et al. 2017; Shamsuzzaman And Biswas 2012; Verdon et al. 2015). Although PADs have never been registered in the EU as veterinary medicinal products for application in fish and crustacean farming, their residues are still found in aquaculture products (EFSA 2019, 2020, 2021, 2022, RASFF 2025). On the other hand, malachite green and methylene blue have been officially used in fish and shrimp farms in some countries, such as Bangladesh (Rasul et al. 2017) and India (Dutta And Bhattacharya 2021). Up to now, PADs have been found in fish (Dubreil et al. 2019; Fallah And Barani 2014; Kwan et al. 2018; Mhungu et al. 2020), frogs (Turnipseed et al. 2012), shrimps (Lian And Wang 2013), and turtle farming (He And Cui 2016; Shen et al. 2019). Next to the commonly illegally used PADs such as leucomalachite green (Dinh et al. 2020; Kaplan et al. 2014; Panapong et al. 2024; Pipoyan et al. 2019), malachite green (Adel et al. 2016; Barani And Tajik 2017), leucocrystal violet (Gammoh et al. 2019), crystal violet (Amelin et al. 2017; Azarkohan et al. 2013; Fei et al. 2022; Gammoh et al. 2019), also other dyes such as azure A and azure B (Xu et al. 2012), brilliant green (Bahrami et al. 2021), methylene blue (Xu et al. 2009), rhodamine 6G (Park et al. 2020), and Victoria pure blue BO (Dubreil et al. 2019) have been detected in farmed fish (Table 2).

**Table 2:** List of the PADs determined in aquaculture and fishery

Class of dye	Dye name	Type of sample	Analytical method*	Limit of detection [µg/l or µg/kg]	Limit of quantitation [µg/l or µg/kg]	Concentration determined [µg/l or µg/kg]	Country of sample origin	References
**Aquaculture water**								
Triphenylmethane	Brilliant green	Fish farming water	UV–vis spec	15	ND**	99–195	Iran	Tavallali and Ostovar (2009)
		Fish pond water	UV–vis/DAD spec	0.55		10		Es'haghi et al. (2011)
		Fish breeding pool water	UV–vis spec	2.6	8.6	33–50		Khani and Irani (2020)
	Crystal violet	Fish farming water (coastal sea)	HPLC–DAD	0.1	0.35	0.52	China	Lian and Wang (2013)
		Fish farming water (seawater)				0.92		
		Fish farming water	MS	0.16	0.38	0.92–0.95		Cao et al. (2024)
			DAD spec	9	ND	51		Yu et al. (2015)
	Malachite green	Fish farm effluent	UV–vis spec	ND		0.0057–0.384	Iran	Khodabakhshi and Amin (2012)
		Fish farming water		1.2		10.5–12.9		Pourreza and Elhami (2007)
				0.2		279		Shariati-Rad and Haghparast (2020)
				3.2		7.6		Farhadi et al. (2010)
			DAD spec	17		83	China	Yu et al. (2015)
			Raman spectroscopy	0.1		0.1		Zhang et al. (2018)
			HPLC–DAD		0.3	7.6	Iran	Maleki et al. (2012)
	∑ = malachite green + leucomalachite green		UV–vis spec	0.28	ND	30.6		Afkhami et al. (2010)
	Malachite green	Fish storage water	LC–MS/MS	25	80	2 020	Saudi Arabia	Hakami et al. (2021)
		Pet fishpond water	HPLC–UV	5	ND	0.67–1.5	China	Li et al. (2008)
**Aquaculture sediment**								
Triphenylmethane	Leucomalachite green	Pond sediment	HPLC–MS/MS	1.6	3.0	6–52	Austria	Weiß and Schmutzer (2010)
	Malachite green	Bottom sediment of fish farming pool	UV–vis spec	0.2	ND	2 342	Iran	Shariati-Rad And Haghparast 2020)
		Bottom sediment of fish farming pond	HPLC–MS/MS	1.8	3.6	< 3.6–140	Austria	Weiß and Schmutzer (2010)
**Fishery and aquaculture products**								
Phenothiazine	Azure A	Fish	UPLC-MS/MS	0.3	1	1.62	China	Xu et al. (2012)
	Azure B					3.27		
	Methylene blue	Fish, shrimp	LC–MS/MS	0.1	0.5	1–20		Xu et al. (2009)
Triphenylmethane	Brilliant green	Fish	UV–vis spec	0.28	0.94	5	Iran	Bahrami et al. (2021)
	Crystal violet	Caviar	HPLC-HR-TOF–MS	ND	0.01	5.3	Russia	Amelin et al. (2017)
Triphenylmethane	Crystal violet	Fish	HPLC–DAD	0.05	0.17	0.36	China	Lian and Wang (2013)
			LC–MS/MS	0.02	0.09	0.362–41.34	imported to Jordan from Vietnam, United Arab Emirates, China	Gammoh et al. (2019)
			UPLC-MS/MS	0.3	1	7.15	China	Xu et al. (2012)
			UV–vis	1.3	ND	1.6	Iran	Azarkohan et al. (2013)
		Shrimp	HPLC–DAD	0.05	0.17	0.27	China	Lian and Wang (2013)
		Sea snail	UPLC-UV	0.35	1.15	10.6		Fei et al. (2022)
	Leucocrystal violet	Fish	LC–MS/MS	0.02	0.09	0.178–10.58	imported to Jordan from Vietnam, China	Gammoh et al. (2019)
	Leucomalachite green	Fish		0.28	0.37	0.6–1	Turkey	Kaplan et al. (2014)
				0.24	0.31	0.4–1.7		
				ND	ND	0.05–0.88	Malaysia	Kwan et al. (2018)
			UPLC-MS/MS	0.3	1	16.3	China	Xu et al. (2012)
				1	ND	1–251		Mhungu et al. (2020)
			HPLC-HR-TOF–MS	ND	0.3	0.9	Russia	Amelin et al. (2017)
			HPLC–MS/MS		ND	0.3–3.8	Austria	Weiß and Schmutzer (2010)
			dual-ICTS	0.197		0.9	Thailand	Panapong et al. (2024)
		∑ = fish + shrimp	UHPLC-MS/MS	0.003 0.01		0.003–0.9	Imported to Canada from China, Thailand	Dinh et al. (2020)
		Turtle	UPLC-MS/MS	0.18	0.60	0.65–0.92	China	Shen et al. (2019)
	∑ = leucomalachite green + malachite green	Fish	LC–MS/MS	0.5	ND	0.3–4.8	Armenia	Pipoyan et al. (2020)
	Malachite green		ELISA	0.3		0.35–7.12	Iran	Barani and Tajik (2017)
			HPLC-HR-TOF–MS	ND	0.02	2.5	Russia	Amelin et al. (2017)
			LC–MS		ND	265–1663	Iran	Khodabakhshi and Amin (2012)
			LC–MS/MS	0.04		0.036	France	Dubreil et al. (2019)
				ND		0.33–3.51	Malaysia	Kwan et al. (2018)
			HPLC–DAD	0.16	0.39	0.3–146.1	Iran	Fallah and Barani (2014)
				ND	0.3	20–890	Iran	Adel et al. (2016)
			HPLC-FLD	0.5	0.8	1.1–3.3	China	He and Cui (2016)
			HPLC–MS/MS	ND	ND	0.1	Austria	Weiß and Schmutzer (2010)
		Fish	dual-ICTS	0.168	ND	0.85–1.01	Thailand	Panapong et al. (2024)
			UV–vis spec	0.2		1711	Iran	Shariati-Rad and Haghparast (2020)
		Snail	UPLC-UV	0.5	1.65	21.6	China	Fei et al. (2022)
		Turtle	HPLC-FLD	0.5	0.8	0.6–0.7		He and Cui (2016)
Triphenylmethane	Malachite green	Turtle	UPLC-MS/MS	0.16	0.52	0.6–1.1	China	Shen et al. (2019)
	Victoria pure blue BO	Fish	LC–MS/MS	0.06	ND	0.066	France	Dubreil et al. (2019)
Triphenylmethane/xanthene	Crystal violet, malachite green, Victoria pure blue BO/rhodamine 6G	Fish		2		0.1–1.4	Korea	Park et al. (2020)

^*^Analytical methods: *dual-ICTS* dual immunochromatographic test strip, *ELISA* enzyme-linked immunosorbent assay, *DAD spec* spectrophotometer with diode array detector, *HPLC-DAD* high performance liquid chromatography with diode array detector, *HPLC-FLD* high-performance liquid chromatography with fluorescence detector, *HPLC-HR-TOF-MS* high-performance liquid chromatography-high resolution-time-of-flight-mass spectrometry, *HPLC-MS/MS* high-performance liquid chromatography with tandem mass spectrometry, *HPLC-UV* high-performance liquid chromatography with ultraviolet detector, *LC-MS* liquid chromatography-mass spectrometry, *LC-MS/MS* liquid chromatography-tandem mass spectrometry, *MS* mass spectrometer, Raman spectroscopy, *UPLC-MS/MS* ultra high-performance liquid chromatography-tandem mass spectrometer, *UPLC-UV* ultra high-performance liquid chromatography with ultraviolet-visible spectrophotometry, *UV-vis/DAD* ultraviolet-visible with diode array detector, *UV-vis spec* ultraviolet-visible spectrophotometry. ND** no data

The important issue is the determination of PADs in aquaculture sediments. So far, malachite green (Shariati-Rad And Haghparast 2020; Weiß And Schmutzer 2010) and its metabolite leucomalachite green (Weiß And Schmutzer 2010) have been found in fish farming sediments in a concentration of < 3.6–2 342 µg/kg (Table 2). Weiß and Schmutzer (2010) analysed water, sediment, and fish samples from 15 fish farms, including 8 trout farms and 7 carp farms. PADs could not be detected in any of the water samples, while they were found in fish (0.1–3.8 µg/kg) and sediment (< 1.6–140 µg/kg) from 6 and 5 fish farms, respectively. Only if a low concentration of malachite green in fish was found (0.1 µg/kg), no dyes were present in the sediment. Interestingly, malachite green and leucomalachite green were also found in sediments downstream of the fish farm (where PADs were determined in fish and sediments) but were not detected in the sediments upstream of the fish farms. (Weiß And Schmutzer 2010). In this case, malachite green most likely did not enter the fish farm through the influent. It shows sediments act as an “integrating long-term memory” accumulating PADs; compared to fish or water samples, their presence can be detected for a longer period. In this way, contaminated sediments may pose an indirect source of dyes in the aquatic environment. Also, a study conducted by Mukherjee et al. (2024) showed that in natural settings, almost 64% of crystal violet from water undergoes a transition to sediment.

Up to now, PADs such as brilliant green (Bahrami et al. 2021; Es'haghi et al. 2011; Tavallali And Ostovar 2009), crystal violet (Cao et al. 2024; Lian And Wang 2013; Yu et al. 2015), and malachite green (Afkhami et al. 2010; Farhadi et al. 2010; Hakami et al. 2021; Khodabakhshi And Amin 2012; Li et al. 2008; Maleki et al. 2012; Pourreza And Elhami 2007; Zhang et al. 2018) belonging to triphenylmethane dyes have been determined in different fish farming water (breeding pool water, effluent, storage, or pet fish water) (Table 2). Malachite green (0.0057–2 020 µg/l) poses more than 60% of cases described. After malachite green, the next illegally used dye is brilliant green in concentration (5–50 µg/l) and crystal violet (0.52–0.95 µg/l). It is worth highlighting that crystal violet was found in water from fish farming localised in an area of coastal sea. The presented data in Table 2 shows that problems with illegal applications of PADs in aquaculture still exist.

Most inland fish farming is based on open or semi-open water systems. If fish farmers use PADs illegally, they add them directly to fish tanks as a mono- or multicomponent of treatment baths (to dissolve in water) (Sudova et al. 2007; Zhang et al. 2018) or by fish feed containing PADs (Li et al. 2008). Thus, it should be noted that PAD concentrations added illegally to fish farming water are higher than that determined in water due to dilution. It also indicates that fish farms may directly contaminate local water and bottom sediment with dyes and others located below fish farms and human water resources (Tkaczyk et al. 2020). On the other hand, if PADs are present in the environmental water (Table 3), these dyes could contaminate aquaculture products that act as environmental filters and absorb PADs.

**Table 3:** PADs determined in different types of environmental samples

Class of dye	Dye name	Type of water	Analytical method	Limit of detection [µg/l or µg/kg]	Limit of quantitation [µg/l or µg/kg]	Concentration determined [µg/l or µg/kg]	Country of sample origin	References
**Water**								
Phenothiazine	Methylene blue	Seawater	UV–vis spec	0.6	2	30	Iran	Badiee et al. (2019)
Triphenylmethane	Brilliant green	Pool water		300	ND	500		Bagtash and Zolgharnein (2018)
		River water						
		Seawater		0.3		5.4–6.3		Seraj et al. (2022)
	Crystal violet	Dam water	UPLC-MS/MS	0.003	0.01	0.0209	Poland	Tkaczyk-Wlizlo and Mitrowska (2023)
		Lake water				0.0122		
		Pool water	UV–vis spec	4.8	ND*	0.9	China	An et al. (2010)
		River water	HPLC–UV	0.03		0.049–0.870		Zhang et al. (2012b)
	Malachite green	Lake water	LC–MS/MS	25	80	430	Saudi Arabia	Hakami et al. (2021)
		Pool water	HPLC–UV	0.080		0.173	China	Gao et al. (2013)
			UV–vis spec	2.9		2.1		An et al. (2010)
		River water	HPLC–UV	0.086	ND	0.20–0.62		Zhang et al. (2012b)
			UV‐vis spec	82		0.32–0.71	Malaysia	Azni et al. (2024)
				300		600	Iran	Bagtash and Zolgharnein (2018)
		Seawater	HPLC	0.083		1.4–3.5		Hashemi et al. (2019)
	Methyl violet	River water	UV‐vis spec	2.20	7.34	3.82	Pakistan	Ul Haq et al. (2023)
	Methyl violet 2B	Dam water	UPLC-MS/MS	0.003	0.01	0.0571	Poland	Tkaczyk-Wlizlo and Mitrowska (2023)
		Lake water				0.0274		
Xanthene	Rhodamine b	Dam water				0.0594		
		River water	HPLC-FLD	0.0005	0.0015	0.0048	Taiwan	Chiang et al. (2011)
		Seawater	HPLC	0.010	ND	0.60–1.20	Iran	Hashemi et al. (2019)
	Rhodamine 6G	River water	HPLC-FLD	0.0001	0.0003	0.0013	Taiwan	Chiang et al. (2011)
			UV–vis spec	0.9	3	76.6	Iran	Badiee et al. (2019)
		Seawater				23		
**Sediment and soil**								
Triphenylmethane	Leucocrystal violet	Soil	GC	ND	ND	2,000,000	USA	Nelson and Hites (1980)
	Leucomalachite green	River sediment				100		
		Sediment	HPLC–MS/MS			16–19	Austria	Weiß and Schmutzer (2010)
Triphenylmethane	Leucomalachite green	Soil	GC	ND	ND	1,000,000–2,000,000	USA	Nelson and Hites (1980)
	Malachite green	River sediment	HPLC–MS/MS	1	3	4.7–25.6	Germany	Ricking et al. (2013)
		River suspended particulate matter	HPLC–MS/MS	1	3	12.7–543	Germany	Ricking et al. (2013)
		Sediment		ND	ND	6–140	Austria	Weiß and Schmutzer (2010)
**Wild aquatic animals**								
Triphenylmethane	Brilliant green, Victoria blue R, Victoria blue B	European eel (*Anguilla anguilla*)	UPLC-MS/MS	< 0.01	0.25	Detected on level < LOQ	Belgium	Belpaire et al. (2015), Reyns et al. (2014)
	∑ = crystal violet + leucocrystal violet					0.12–2.6		
			HPLC–MS/MS	0.01 0.005	0.02 0.01	0.06–6.71	Germany	Schuetze et al. (2008a)
	Leucomalachite green	Sea snail (*Babylonia lutosa*)	UPLC–MS/MS	ND	1	2.48	China	Pan and Han (2023)
	∑ = malachite green + leucomalachite green	European eel (*Anguilla anguilla*)	HPLC–MS/MS	0.02 0.01	0.04 0.02	0.044–0.765	Germany	Schuetze et al. (2008b)
			UPLC-MS/MS	< 0.01	0.25	0.12–9.96	Belgium	Belpaire et al. (2015), Reyns et al. (2014)

^*^Analytical methods: *GC* gas chromatography, *HPLC* high-performance liquid chromatography, *HPLC-FLD* high-performance liquid chromatography with fluorescence detector, *HPLC-MS/MS* high-performance liquid chromatography with tandem mass spectrometry, *HPLC-UV* high-performance liquid chromatography with ultraviolet detector, *LC-MS/MS* liquid chromatography-tandem mass spectrometry, *UPLC-MS/MS* ultra high-performance liquid chromatography-tandem mass spectrometer, *UV-vis spec* ultraviolet-visible spectrophotometry.**ND no data

## Treatment of PAD-containing wastewater in WWTPs

**Fig. 2:**
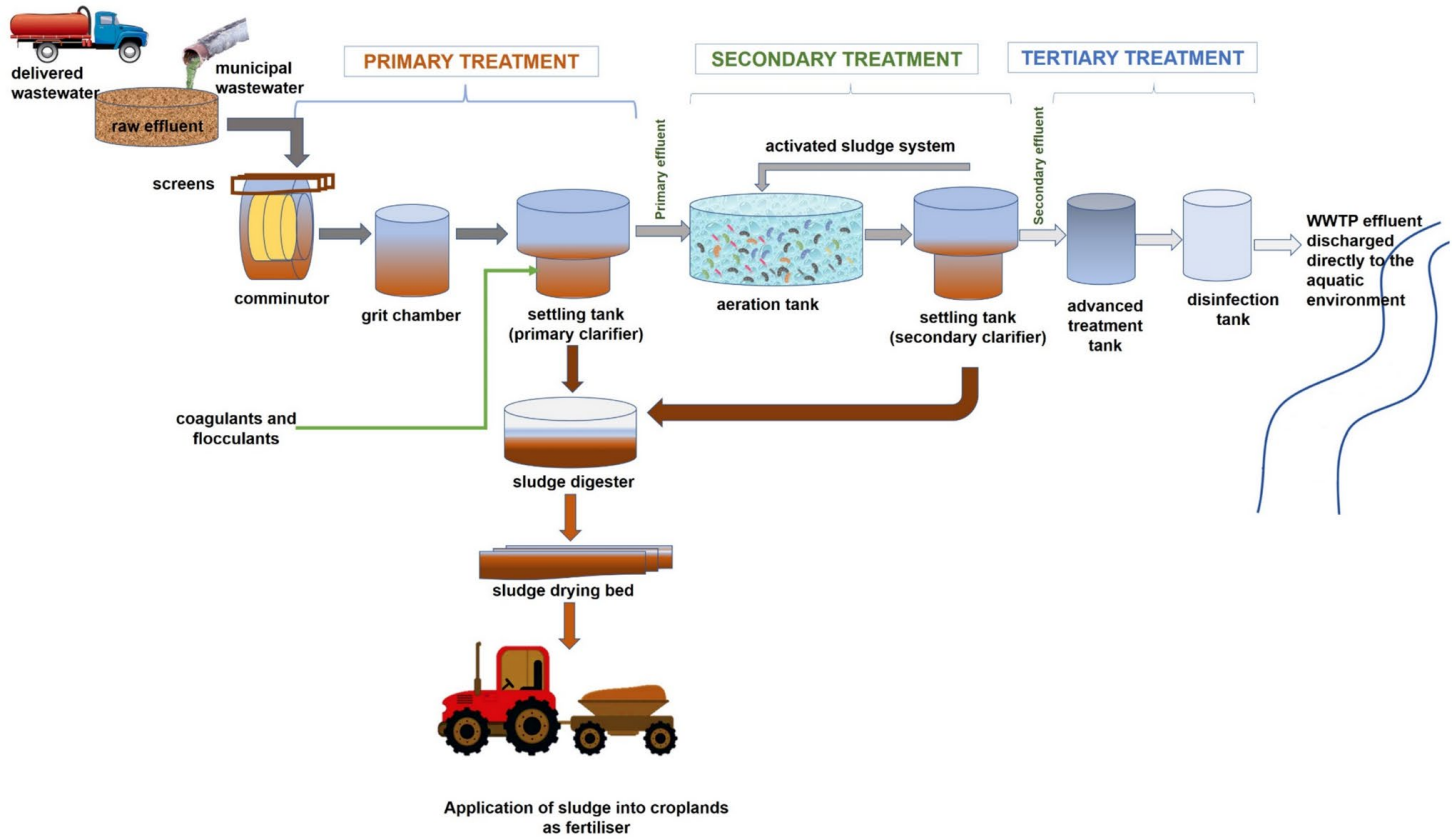
Wastewater treatments in WWTP

Depending on the type of wastewater, WWTPs apply primary, secondary, and tertiary treatment processes (Fig. 2) using different biological, chemical, and physical methods to degrade contained inorganic and organic compounds, heavy metals, and toxic substances to finally obtain acceptable pollutant concentration limits before they are discharged into environmental water (Al-Tohamy et al. 2022; Sathya et al. 2022; Zaharia And Suteu 2012). Due to a lack of appropriate legislation or norms and, as a result, limits and analytical methods, the most commonly controlled parameters in WWTPs are 5-day biochemical oxygen demand (BOD_5_), chemical oxygen demand (COD), and total organic carbon (TOC). This approach may be insufficient to verify the efficiency of PADs’ degradation in wastewater (Rusell 2019). In dye-containing wastewater, colour is often considered the only indication of contamination, not actual PAD concentrations. This is why, after different treatments, including the decolourisation step, dyes and their derivatives are still present in treated wastewater effluents discharged directly to the aquatic environment (Alsukaibi 2022).

Primary treatment is based on removing different materials, such as sticks, grit, and garbage from wastewater, which may inhibit the operation of downstream processes. In the beginning, wastewater flows across coarse and fine screens. Next, the comminutor crumbles solid parts still present in the wastewater, and liquid matter undergoes through a grit chamber to a settling tank (primary clarifier) (Fig. 2) (Iyare et al. 2020; Sonune And Ghate 2004).

Sedimentation occurs in clarifiers where suspended particles such as silts and clay are settled. To increase the process’s effectiveness, flocculants and coagulants such as alum, ferric chloride, ferric sulphate, or lime are commonly added. Afterwards, the settled particles are collected as sludge and transferred to a sludge digester. Finally, sludge is seated on a sludge drying bed where, after drying, it is applied to lands or used to produce building materials. The appropriate disposal of sludge poses an environmental problem and is one of the biggest challenges of WWTPs (Ghaly et al. 2013; Yang et al. 2015).

The next step is a secondary treatment based on biological methods where microorganisms such as algae, bacteria, and fungi in an aeration tank utilise the organic matter in wastewater. Different biological processes are applied during secondary treatment, including biofiltration, biological oxidation, biosynthesis, and activated sludge. After the decomposition of organic matter by microorganisms from the activated sludge system, wastewater flows to a settling tank (secondary clarifier), where dense biomass undergoes sedimentation (Samer 2015).

The tertiary treatment removes additional organic and suspended solids or toxic compounds from wastewater. Many physicochemical methods are used during this step, such as adsorption, ion exchange, membrane filtration, ozonation, reverse osmosis, and advanced oxidation processes (AOPS). After applying the advanced techniques, wastewater undergoes disinfection and, finally, as effluent, is discharged into the local aquatic environment (Fig. 2) (Sathya et al. 2022; Zagklis And Bampos 2022). The high amount of dried sludge causes its disposal to be a problem. Currently, dried sludge undergoes incineration or is used as fertiliser in agriculture (Lamastra et al. 2018; Samer 2015).

PADs as organic synthetic dyes have been designed to be stable to light, oxidising, and microbial agents to maintain colour on items for longer. However, the advantages of PADs make them very difficult to degrade (Elumalai And Muthuraman 2013; Shaffiqu et al. 2002).

There is no consistent approach to treating dye-containing wastewater because they differ in physicochemical properties. Moreover, verifying what types of dyes are in wastewater is problematic due to the lack of analytical methods for determining many different PADs in one approach.

PADs determined in wastewater and examples of different methods from primary, secondary, and tertiary WWTP treatments of PADs in wastewater are presented in Tables 4 and 5, respectively. Depending on the dye, various approaches have been tested to degrade individual dye effectively.

**Table 4:** PADs determined in different types of industrial and municipal wastewater

Class of dye	Dye name/acronym	Type of wastewater	Analytical method	Limit of detection [µg/l]	Limit of quantitation [µg/l]	Concentration determined [µg/l]	References
**Industrial wastewater**							
Azo	Chrysoidine	Textile industry wastewater	HPLC–DAD	0.5	ND	3.4–11.4	Qi et al. (2016)
Phenothiazine	Methylene blue	Laundry wastewater	UPLC-MS/MS	0.3	0.9	360	Khan et al. (2014)
				8	25	390–430	Hakami et al. (2020)
		Microbiology laboratory	UV–vis spec	ND	ND	138,000	Aziz et al. (2021)
		Medical lab wastewater				33,000	Aziz and Karim (2019)
						60,000–125,000	Salh et al. (2019)
		Paper industry wastewater	UPLC-MS/MS	0.3	0.9	540	Khan et al. (2014)
		Printing industry wastewater				830	
				8	25	1 930	Hakami et al. (2020)
		Plastics industry	UV–vis spec	0.6	2	82.5	Badiee et al. (2019)
		Textile industry wastewater	UPLC-MS/MS	0.3	0.9	1080	Khan et al. (2014)
				8	25	4020–5560	Hakami et al. (2020)
Triphenylmethane	Brilliant green	Fish aquarium wastewater	UV–vis spec	47	ND	1220	Damirchi et al. (2019)
		Textile industry wastewater				3 220	
	Crystal violet	Laundry wastewater	UPLC-MS/MS	8	25	270–380	Hakami et al. (2020)
		Printing industry wastewater				2090	
		Textile industry wastewater	UV–vis spec	50	ND	1100	Kamaruddin et al. (2017)
			UPLC-MS/MS	0.001		0.015	Methneni et al. (2021)
			UV–vis spec	0.82		1428	Zhang et al. (2012a)
			UPLC-MS/MS	8	25	3890–4360	Hakami et al. (2020)
	Malachite green	Laundry wastewater		0.1	0.4	1 320	Khan et al. (2019)
			LC–MS/MS	25	80	390	Hakami et al. (2021)
		Leather industry				2560	
		Paper industry wastewater	UPLC-MS/MS	0.1	0.4	620	Khan et al. (2019)
		Printing industry wastewater				790	
		Textile industry wastewater				1680	
Triphenylmethane	Malachite green	Textile industry wastewater	UV–vis spec	ND	ND	105,000	Lam et al. (2018)
		Wastewater***	UV–vis spec	0.055	ND	0.0025	Ullah et al. (2023)
Xanthene	Rhodamine B	Laundry wastewater	UPLC-MS/MS	8	25	320–410	Hakami et al. (2020)
		Textile industry wastewater	HPLC–DAD	0.3	ND	2.9–13.9	Qi et al. (2016)
			UV–vis spec	0.8		13.1	Unsal et al. (2014)
			UPLC-MS/MS	8	25	1 030–1920	Hakami et al. (2020)
			UV–vis spec	1.05	ND	28.62	Taziki et al. (2012)
				0.8		13.1	Unsal et al. (2014)
			HPLC–DAD	0.3		2.9–13.9	Qi et al. (2016)
		Plastics industry wastewater	UV–vis spec	2.4	7.9	201.8	Biparva et al. (2010)
				0.9	3	115.4	Badiee et al. (2019)
		Textile industry wastewater		2.4	7.9	112.9	Biparva et al. (2010)
				50	ND	1000	Kamaruddin et al. (2017)
				0.9	3	55.8	Badiee et al. (2019)
**Municipal wastewater**							
Phenothiazine	Methylene blue	Municipal wastewater	UV–vis spec	60	200	1 530	Razmara et al. (2011)
Triphenylmethane	Crystal violet	WWTP effluent	UPLC-MS/MS	0.003	0.01	0.023	Tkaczyk-Wlizlo et al. (2022)
			HPLC–UV	0.030	ND	0.317	Gao et al. (2013)
	Malachite green	Aquarium water	UV–vis spec	0.2		806–1102	Shariati-Rad and Haghparast (2020)
				3.6		110	Aydin et al. (2017)
		WWTP effluent	HPLC–UV	0.080		0.143	Gao et al. (2013)
	Methyl violet 2B		UPLC-MS/MS	0.003	0.01	0.017	Tkaczyk-Wlizlo et al. (2022)
Xanthene	Rhodamine B	Municipal wastewater	HPLC-FLD	0.0005	0.0015	0.037–0.062	Chiang et al. (2011)
		WWTP effluent	UPLC-MS/MS	0.003	0.01	0.027–0.043	Tkaczyk-Wlizlo et al. (2022)
	rhodamine 6G	municipal wastewater	HPLC-FLD	0.0001	0.0003	0.0007	Chiang et al. (2011)

^*^Analytical methods: *HPLC-DAD* high-performance liquid chromatography with diode array detector, *HPLC-FLD* high-performance liquid chromatography with fluorescence detector, *HPLC-UV* high-performance liquid chromatography with ultraviolet detector, *LC-MS/MS* liquid chromatography-tandem mass spectrometry, *UPLC-MS/MS* ultra high-performance liquid chromatography-tandem mass spectrometer, *UV-vis spec* ultraviolet-visible spectrophotometry. **ND no data.***Type of wastewater not determined

**Table 5:** Examples of different levels of wastewater treatments containing PADs

Class of dye	Pharmacologically active dye	Level of WWTP treatment	Methods	Components	Maximal effectiveness of dye removes [%]	Reference
Phenothiazine	Methylene blue	Primary	Coagulation	Aluminium sulphate-*Aloe vera* magnesium sulphate-*Aloe vera*	50–55 60–70	Lee et al. (2015)
				Laterite soil	99.6	Lau et al. (2015)
			Electro-coagulation	Iron and aluminium electrode	98–100	Alizadeh et al. (2015)
Triphenylmethane	Ethyl violet		Flocculation	Phosphorylated birchwood xylan	> 95	Liu et al. (2018b)
	Malachite green			Carboxylate-rich magnetic chitosan	98.3	Liu et al. (2018a)
	Pararosaniline		Electro-coagulation	Inter-electrode	99	Nandi and Patel (2013)
Xanthene	Rhodamine 6G			Iron-based electrode	98	Zaleschi et al. (2014)
Azo	Chrysoidine	Secondary	Biodegradation	Peroxidase enzyme from plant *Ipomoea palmata* and *Saccharum spontaneum*	44 70	Shaffiqu et al. (2002)
			Decolourisation	yeast strain *Candida curuvata* AN723	97.9	Kakuta et al. (1998)
Phenothiazine	Azure B			Bacteria *Streptomyces ipomoeae*	13.53	Blanquez et al. (2019)
				Bacteria *Bacillus* sp.* LD003*	90	Bandounas et al. (2013)
	Methylene blue		Biosorption	Fungi *Saccharomyces cerevisiae*	96	Yu et al. (2009)
Triphenylmethane	Brilliant green		Decolorisation and biodegradation	Fungi *Aspergillus* sp.	100	Kumar et al. (2012)
			Biodegradation	Peroxidase enzyme from plant *Ipomoea palmata*	54	Shaffiqu et al. (2002)
				Bacteria *Klebsiella* strains	81.4	Tiwari et al. (2022)
	Crystal violet		Decolorisation and biodegradation	bacteria *Bacillus sp*	100	Ayed et al. (2009)
			Biodegradation	Bacteria *Pseudomonas putida*	80	Chen et al. (2007)
				Peroxidase enzyme from plant *Ipomoea palmata*	36.3	Shaffiqu et al. (2002)
			Decolourisation	Bacteria *Streptomyces bacillaris*	86.6	Adenan et al. (2022)
	Malachite green		Decolourisation and biodegradation	Bacteria *Klebsiella aerogenes* S27	90 97	Shang et al. (2019)
				Bacteria *Pseudomonas* sp. strain YB2	90.4 99.9	Tao et al. (2017)
			Decolourisation	Bacteria *Streptomyces bacillaris*	94.7	Adenan et al. (2022)
	Methyl violet				91.8	
Acridine	Acriflavine	Tertiary	Membrane filtration	Poly(diallyldimethylammonium chloride)/graphene oxide	44	Gyenes et al. (2020)
Phenothiazine	Azure A		Adsorption	Snail shell modified by melamine formaldehyde polymer	93.9	Al-Shemary et al. (2020)
	Azure B		Adsorption	Rice husk	90–97	Duraisamy et al. (2015)
Phenothiazine	Methylene blue	Tertiary		Coconut fibre husks	98.3	Wong et al. (2013)
				Indian Rosewood sawdust	97.1	Garg (2004)
				Sheep wool fibre cotton fibre	94.3 97	Khan et al. (2005)
			Photocatalytic degradation	Co3O4/ZnO nanocomposite	86	Hassanpour et al. (2017)
Triphenylmethane	Brilliant green		Adsorption	*Lawsonia inermis* seed	93	Ahmad and Ansari (2020)
	Crystal violet		Activated carbon	Activated carbon based on risk husk	93.5	Mohanty et al. (2006)
			Adsorption	Almond husk *Bambusa tulda*	96.9 > 95	Bhanuprakash and Belagali (2017) Laskar and Kumar (2017)
			Photocatalytic degradation	ZnO	93	Sacco et al. (2018)
				ZnO–Co	100	Fahoul et al. (2022)
	Malachite green		Activated carbon	Groundnut shell	96	Malik et al. (2007)
			Adsorption	Coconut fibre husks	99	Wong et al. (2013)
				Neem sawdust	83.6	Khattri and Singh (2009)
			Adsorption	*Pinus patula* wood	99.7	Rubio-Clemente et al. (2021)
			Advanced oxidation	Ultrasonic electrochemical process	94.9	Ren et al. (2021)
			Membrane filtration	Polymer inclusion membrane	98	Ling and Mohd Suah (2017)
	Pararosaniline		Adsorption	Almond husk	95.6	Bhanuprakash and Belagali (2017)
	Victoria blue				95	
Xanthene	Rhodamine B		Active carbon	Active carbon based on lemon citrus peel	99.3	Sharifzade et al. (2017)
				Active carbon based on rice husk	95.3	Ding et al. (2014)
			Adsorption	*Argemone mexicana* weed Modified Alpha Alumina Nanoparticles	70 95.2	Khamparia and Jaspal (2016) Yen Doan et al. (2020)
			Membrane filtration	Bulk liquid membrane (phenol, xylene)	86	Elumalai and Muthuraman (2013)
			Photocatalytic degradation	Co_3_O_4_/ZnO nanocomposite	91	Hassanpour et al. (2017)

Among the primary treatment methods, coagulation (Lau et al. 2015; Lee et al. 2015), electrocoagulation (Alizadeh et al. 2015; Nandi And Patel 2013; Zaleschi et al. 2014), and flocculation (Liu et al. 2018a, 2018b) have been described (Table 5). Application of coagulation-flocculation during primary treatment of wastewater containing dyes is common. Coagulation removes dissolved and colloidal substances, and flocculation helps particles form larger parts to finally undergo sedimentation (Lee et al. 2015; Liu et al. 2018a). The most popular are chemical coagulants such as aluminium sulphate or ferric chloride. Unfortunately, their applications cause obtained sludge to be challenging to degrade and pose additional environmental problems following wastewater disposal. Hence, the usage of natural coagulants such as laterite soil (Lau et al. 2015) or hybrid coagulants such as magnesium sulphate-*Aloe vera* (Lee et al. 2015) might be a better solution (Table 5).

The aim of secondary treatment of wastewater is decolourisation and biodegradation of PADs using microorganisms such as bacteria belonging to the different types of species *Bacillus* (Ayed et al. 2009; Bandounas et al. 2013), *Klebsiella* (Shang et al. 2019; Tiwari et al. 2022), *Pseudomonas* (Chen And Lu 2007; Tao et al. 2017), and *Streptomyces* (Adenan et al. 2022; Blanquez et al. 2019) and fungi *Aspergillus* (Kumar et al. 2012) and *Candida* (Kakuta et al. 1998), as well as baker’s yeast *Saccharomyces cerevisiae* (Yu et al. 2009) (Table 5). Among different species of microorganisms, the highest rate of dye degradation has been obtained using the fungi *Aspergillus* (brilliant green; 100%) (Kumar et al. 2012) and bacteria *Klebsiella aerogenes* S27 (malachite green; 97%) (Shang et al. 2019). Also, the activity of plant-derived enzymes has been tested in dye biodegradation, but their effectiveness is less than that of microbial enzymes. For instance, the application of peroxidase from *Ipomoea palmata* allows the degrading of only 54% of brilliant green and 44% of chrysoidine, while peroxidase from *Saccharum spontaneum* was effective in 70% in the case of chrysoidine removal (Shaffiqu et al. 2002).

Many scientists are trying to improve or design new tertiary methods as the last link of wastewater treatment. Up to now, methods such as adsorption (Al-Shemary et al. 2020; Duraisamy et al. 2015; Wong et al. 2013), activated carbon (Ding et al. 2014; Mohanty et al. 2006; Sharifzade et al. 2017), advanced oxidation (Ren et al. 2021), membrane filtration (Gyenes et al. 2020; Ling And Mohd Suah 2017), and photocatalytic degradation (Fahoul et al. 2022; Hassanpour et al. 2017; Sacco et al. 2018) have been used (Table 5).

The applications of different plant-derived materials as adsorbents to remove dyes have become popular, especially the usage of agriculture by-products such as almond husk (Bhanuprakash And Belagali 2017), coconut fibre husk (Wong et al. 2013), cotton fibre (Khan et al. 2005), neem sawdust (Khattri And Singh 2009), rice husk (Duraisamy et al. 2015; Mohanty et al. 2006), and biomass of *Pinus patula* wood (Rubio-Clemente et al. 2021), *Argemone mexicana* weed (Khamparia And Jaspal 2016), *Lawsonia inermis* (Ahmad And Ansari 2020), and *Dalbergia sissoo* (Garg 2004). The advantage of this approach is the usage of natural, cheap, and available materials, most of which have a high rate of dye removal (> 95%) (Bhanuprakash And Belagali 2017; Laskar and Kumar 2017; Rubio-Clemente et al. 2021; Wong et al. 2013). Also, the usage of nanoparticles in dye adsorption was tested (Yen Doan et al. 2020). However, it is not a common approach (Table 5).

Another method of tertiary wastewater treatment is the usage of activated carbon also based on agricultural waste such as groundnut shell (Malik et al. 2007), lemon citrus peel (Sharifzade et al. 2017), or rice husk (Ding et al. 2014; Mohanty et al. 2006). For dye removal from wastewater also, additional methods are applied, such as advanced oxidation (Ren et al. 2021), membrane filtration (Elumalai And Muthuraman 2013; Gyenes et al. 2020; Ling And Mohd Suah 2017), and photocatalytic degradation (Fahoul et al. 2022; Hassanpour et al. 2017; Sacco et al. 2018). However, the abovementioned methods are based mainly on individual dye removal tested in laboratory conditions, not on real wastewater containing different components. Therefore, the actual efficiency of the degradation of PADs will probably be lower. Moreover, there is still no complex approach to treating wastewater containing a mixture of different PADs.

## The sources and occurrence of PADs in the aquatic environment

Because of their pharmacological and colouring properties, PADs are used in the pharmaceutical industry, hospitals, human medicine, and veterinary medicine and in the cosmetic, paper, plastic, printing, textile industries, tanning, and households. The application of PADs in the areas mentioned above, their improper use and disposal, and the wastewater they produced resulted in directed contamination of the environment (Fig. 1) (Tkaczyk et al. 2020). WWTPs collect industrial and municipal wastewater. The primary, secondary and tertiary treatments used in WWTPs are insufficient to degrade all PASs, including PADs present in wastewater. Due to that, WWTPs pose an indirect source of PADs in the environment because generated effluent is directly discharged to open waters, and sewage sludge is used as organic fertilisers in agriculture. Also, the application of sediment from aquaculture as fertiliser, if PADs are used illegally, poses an indirect source of dyes in the environment (Fig. 1).

Among the different reports about chemical contaminants in the aquatic environment, there is little concern about the occurrence of PADs. Up to now, PADs have been found in water, sediment, soil, suspended particulate matter, and wild aquatic animals (Table 3).

### Water

PADs belonging to phenothiazine, triphenylmethane, and xanthene dyes have been found in different environmental waters such as dams (Tkaczyk-Wlizlo And Mitrowska 2023), lakes (Hakami et al. 2021), pools (An et al. 2010; Gao et al. 2013), rivers (Chiang et al. 2011; Ul Haq et al. 2023), and seawater (Badiee et al. 2019; Seraj et al. 2022) in the concentration range from 0.0013 to 600 µg/l (Table 3). Most determined PADs were triphenylmethane dyes with particular regard to malachite green, the most commonly found PAD in environmental water, independently of the type of water bodies. Moreover, malachite green has been found at the highest concentration (600 µg/l) in river water (Bagtash And Zolgharnein 2018). Studies of river water have shown the occurrence of different PADs belonging to triphenylmethane: brilliant green (Bagtash And Zolgharnein 2018), crystal violet (Zhang et al. 2012b), malachite green (Bagtash And Zolgharnein 2018), and xanthene dyes: rhodamine B and rhodamine 6G (Chiang et al. 2011). The observed different PADs occurrence in water may have resulted from dye-containing wastewater degrading slowly; hence, insufficient WWTP treatments caused dye cycling in the aquatic environment (Tkaczyk et al. 2020). Next, dye-contaminated river water flows into the sea. As a result, PADs belonging to phenothiazine: methylene blue (Badiee et al. 2019), triphenylmethane: brilliant green (Seraj et al. 2022) and malachite green (Hashemi et al. 2019), and xanthene: rhodamine B (Hashemi et al. 2019) and rhodamine 6G (Badiee et al. 2019) have also been indicated in seawater (Table 3). Another source of PADs in environmental water is fish farming. The confirmed occurrence of crystal violet in fish farming seawater (0.52–0.92 µg/l) caused the contamination of the aquatic environment (Table 2) (Lian And Wang 2013).

### Sediment and soil

The occurrence of PADs in the aquatic environment was first described in 1980 in the USA by Nelson and Hites (1980). Leucomalachite green and leucocrystal violet were determined in the soil near the waste storage site from a dye factory at 1000–2000 mg/kg concentration. In the same place, leucomalachite green was found in river sediment at 100 µg/kg. Leaching from the dye waste storage site caused sediment contamination with dyes. As a result, aquatic animals, especially fish (in which previously 1-naphthylamine and N-ethyl-N-phenylbenzylamine were found), were exposed to PADs and their derivates by direct contact with contaminated sediment (Nelson And Hites 1980).

Ricking et al. (2013) performed analyses of suspended particulate matter samples collected from various German rivers and sediments obtained from the Spree and Havel rivers in the urban area of Berlin. The obtained results indicated the presence of malachite green in river sediments in the concentration of 4.7–25.6 µg/kg and in suspended sediment in the range of 12.7–543 µg/kg (Table 3) (Ricking et al. 2013).

Malachite green and leucomalachite green were found in river sediments downstream of the fish farm, where the PADs were present in fish and sediments. One hundred meters below the fish farm, sediments contained 140 µg/kg of malachite green and 16–19 µg/kg of leucomalachite green, while sediments sampled 400 m downstream of the pond were contaminated with malachite green at the concentration of 6–8 µg/kg (Table 3) (Weiß And Schmutzer 2010).

### Aquatic animals

The occurrence of PADs in water and sediments leads to their accumulation in the tissue of living aquatic animals. In 2008, the dyes were detected in free-living fish in the Berlin metropolitan area. PADs calculated as ∑ = crystal violet + leucocrystal violet (0.06–6.71 µg/kg) (Schuetze et al. 2008a) and ∑ = malachite green + leucomalachite green (0.044–0.765 µg/kg) (Schuetze et al. 2008b) were found in European eels (*Anguilla anguilla*) (Table 3). *Anguilla anguilla* fish species were selected due to their high natural fat content of around 25% compared to other fish. Moreover, eels take up sediments from the bottom of the rivers during eating. The lipophilicity of the main metabolites of PADs caused their residues to be longer in fatty fish. Therefore, *Anguilla anguilla* is considered a perfect bioindicator in aquatic environmental contamination with PADs (Schuetze et al. 2008b).

Also, analysis of fish collected in 91 locations in Belgium revealed that 77% of the free-living European eels were contaminated with PADs, including Victoria blue B, pure Victoria blue BO, crystal violet, leucocrystal violet, brilliant green, malachite green, and leucomalachite green (Belpaire et al. 2015; Reyns et al. 2014) (Table 3).

Recently, Pan and Han (2023) studied the contamination of marine gastropods with illicit drugs, including malachite green and leucomalachite green. Among 637 samples of sea snails (*Babylonia lutosa*) in 3.54%, leucomalachite green was determined with a concentration up to 2.48 µg/kg. The obtained results indicate that PADs are present in marine areas of southeast China (Pan And Han 2023).

## Occurrence of PADs in wastewater

Despite the pharmacological properties of PADs, the vast majority are used in textile, tannery, paper, plastic, and printing industries, as well as cosmetic and food production due to their colouring potential (Tkaczyk et al. 2020; Verdon And Andersen 2017). Due to colouring or pharmaceutical potentials, the wide range of application areas caused post-productive waste and wastewater contained PADs.

So far, PADs belonging to azo (chrysoidine) (Qi et al. 2016), phenothiazine: methylene blue (Aziz And Karim 2019; Aziz et al. 2021; Khan et al. 2014; Salh et al. 2019), triphenylmethane: brilliant green (Damirchi et al. 2019), crystal violet (Hakami et al. 2020; Kamaruddin et al. 2017; Methneni et al. 2021), malachite green (Hakami et al. 2021; Khan et al. 2019; Lam et al. 2018; Ullah et al. 2023), methyl violet 2B (Tkaczyk-Wlizlo et al. 2022), and xanthene: rhodamine B (Biparva et al. 2010; Taziki et al. 2012; Unsal et al. 2014) and rhodamine 6G (Chiang et al. 2011) dyes have been determined in wastewater from leather, paper, printing, plastic, and textile production as well as in municipal wastewater (Table 4). PADs are also used during biological staining in different types of laboratories. Thus, a phenothiazine dye, methylene blue, has been found in waste from microbiological and medical laboratories (Aziz And Karim 2019; Aziz et al. 2021). Interestingly, different PADs have also been found in laundry wastewater (Hakami et al. 2020, 2021; Khan et al. 2014, 2019). These reports show that low-quality dyeing of clothes causes quick dye loss during washing (Table 4).

Among PADs in wastewater, the majority of them were triphenylmethane dyes, including brilliant green, crystal violet, and malachite green found in textile industry wastewater (0.015–4 360 µg/l) (Table 4). The application of PADs as commonly used textile dyes poses a severe environmental problem because they are resistant to light and microbial potential; hence, PADs degradation is difficult and complex (Shaffiqu et al. 2002). Moreover, there are examples of textile wastewater being discharged directly into the local aquatic environment without any treatment (Lee et al. 2015).

From another chemical class of PADs, phenothiazine dyes, only methylene blue has been found in the concentration range from 82.5 to 125 000 µg/l in wastewater from a wide range of dyes applications: laundry (Khan et al. 2014), medical laboratory (Aziz And Karim 2019), paper (Khan et al. 2014), plastic (Badiee et al. 2019), printing (Hakami et al. 2020), and textile (Hakami et al. 2020) industries. The presented results show that the most often determined PADs were rhodamine B (xanthene dye), followed by malachite green and crystal violet (triphenylmethane dye) in textile wastewater (Table 4).

It should be noted that PADs such as crystal violet (Gao et al. 2013; Tkaczyk-Wlizlo et al. 2022), malachite green (Gao et al. 2013), methyl violet 2B (Tkaczyk-Wlizlo et al. 2022), methylene blue (Razmara et al. 2011), rhodamine B (Chiang et al. 2011; Tkaczyk-Wlizlo et al. 2022), and rhodamine 6G (Chiang et al. 2011) have also been found in municipal wastewater in the concentration range from 0.0007 to 1 530 µg/l (Table 4). The occurrence of PADs in municipal wastewater may result from different reasons, such as inappropriate disposal of drugs containing PADs, home dyeing of clothes/hair, or as a result of colour loss during laundry (Tkaczyk et al. 2020). Also, applying PADs to aquarium animals (Aydin et al. 2017; Damirchi et al. 2019; Shariati-Rad And Haghparast 2020) resulted in the subsequent release of used water into the municipal sewage system. These results indicate that PADs are omnipresent in our daily lives and, hence, present in municipal wastewater.

## The impact of WWTP sludge containing PADs on the total environment

Depending on the type of wastewater and applied treatment processes, sludge contains different components of organic matter and nutrients, which may improve soil’s physical, chemical, and biological properties. Due to that, sludge is gladly applied in agriculture as a fertiliser (Hudcová et al. 2019). The available data indicates that approximately 40–50% of WWTP sludge produced in the European Union is applied to agriculture (Hudcová et al. 2019; Lamastra et al. 2018).

Despite the merit of sludge usage in plant cultivation, it may also contain different hazardous residues and contaminants such as heavy metals, non-steroidal anti-inflammatory drugs, or antibiotics (Buta et al. 2021; Hudcová et al. 2019; Zhang et al. 2020). Currently, only the presence of selected heavy metals (Cd, Cu, Hg, Ni, Pb and Zn) in sludge applied in farmlands is regulated in the EU (Council of the European Commumities 1986). Other components, especially PASs such as antibiotics, steroids or PADs, have not been regulated.

It is worth emphasising that components of sludge introduced to agriculture may accumulate in the soil, permeate the water system, and consequently cause adverse effects on the total environment, which include lithosphere, hydrosphere, biosphere, and anthroposphere.

Up to now, Zhou (2001) has reported the accumulation of different organic dyes (not pharmacologically active) in farmland. As a result, dyes have been found in crops and vegetables such as rice, wheat, soybean, potato, radish, or watermelon from farmlands fertilised by wastewater containing dyes.

The studies confirmed the negative impact of tested dyes on crop cultivation; hence, their application as fertiliser (a component of wastewater or sewage sludge) should be avoided (Ayed et al. 2009; Zhou 2001). It is worth emphasising that selected dyes accumulate in edible parts of plants and pose an environmental risk to human health (Zhou 2001).

## The impact of PADs occurrence in water on the aquatic food web and risk assessment

**Fig. 3:**
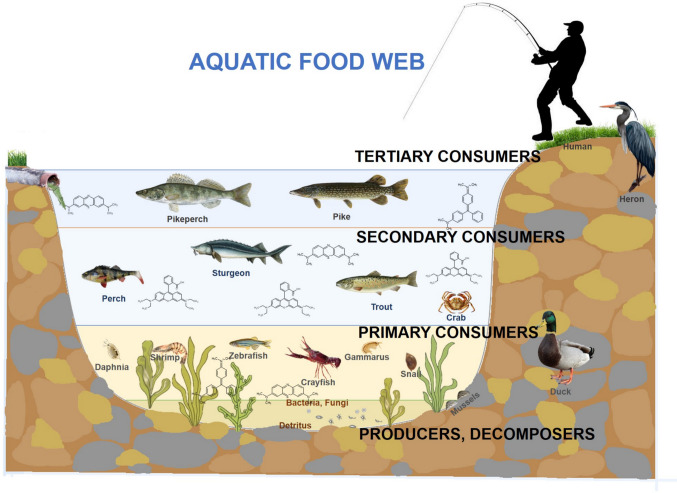
The aquatic food web exposed to PADs

The first sign of dye occurrence in environmental water and wastewater is its colour. This feature is not indifferent to fauna and flora in the water body because colour disturbs light penetration (Al-Tohamy et al. 2022; Alsukaibi 2022; Yu et al. 2009). As a result, limited light availability impacts the photosynthetic activity of water plants and phytoplankton, which pose an essential diet for first-order consumers (Fig. 3) (Malik et al. 2007; Rubio-Clemente et al. 2021; Schaum et al. 2017; Wong et al. 2013). The photosynthesis of phytoplankton is an essential step in the carbon cycle. Almost 40% of the carbon dioxide fixed globally is bonded by phytoplankton, posing the primary productivity of aquatic food webs (Schaum et al. 2017).

The presence of PADs such as auramine, auramine O, crystal violet, leucomalachite green, malachite green, and methylene blue in water impacts producers (green algae, plants) (Adomas et al. 2020; Krishna Moorthy et al. 2021) and decomposers (bacteria, fungi) (Hernando et al. 2007; Nagai 2018) (Table 6). The most toxic PAD was malachite green, especially for bacteria *Vibro fischeri*, with the median effective concentration (EC_50_, _15 min_) = 0.051 mg/l (Hernando et al. 2007) and aquatic fungi, with EC_50, 15 min_ = 0.5–1 mg/l (Bailey And Jeffrey 1989) (Table 6). Based on Commission Directive 93/67/EEC, malachite green is classified as very toxic to aquatic organisms because the EC_50_ value is less than 1 mg/l (Commission of the European Communities 1993).

**Table 6:** PADs with tested toxicity against organisms from different levels of aquatic food web

Food web level	Taxon	Species	Class of PADs	PAD	EC_50_ [mg/l]^1^	LC_50_ [mg/l]^1^				Toxicological effects	Toxicity category	Reference
						24 h	48 h	72 h	96 h			
**Producers, decomposers**	Bacteria	*Vibrio fischeri*	Phenothiazine	Methylene blue	15.55–18.64^2^	ND^6^	ND	ND	ND	ND	*	Fungaroa et al. (2010)
			Triphenylmethane	Malachite green	0.051^2^					Luminescence inhibition		Hernando et al. (2007)
				Leucomalachite green	> 39.9^2^					ND		
	Fungi	*Achlya flagellata*	Triphenylmethane	Crystal violet	1^2^							Bailey and Jeffrey (1989)
				Malachite green								
		*Aphanomyces stellatus*			0.00082^3^							Nagai (2018)
		*Chytriomyces hyalinus*			0.081^3^							
		*Rhizophydium brooksiaum*			0.029^3^							
		*Saprolegnia hypogyna*		Crystal violet	0.75^2^							Bailey and Jeffrey (1989)
				Malachite green	0.5^2^							
		*Sporobolomyces roseus*			0.17^3^							Nagai (2018)
		*Tetracladium setigerum*			0.066^3^							
	Green algae	*Chlorella pyrenoidosa*	Triphenylmethane	Malachite green	ND					Inhibition growth, chromosome aberrations		Kanhere et al. (2014)
		*Chlorella vulgaris*	Phenothiazine	Methylene blue	17.45					ND	II	Krishna Moorthy et al. (2021)
		*Raphidocelis subcapitata*	Triphenylmethane^7^	Auramine	0.3^5^						I	de Jesus Azevedo et al. (2021)
				Auramine O	0.8							
		*Spirulina platensis*	Phenothiazine	Methylene blue	3.77						II	Krishna Moorthy et al. (2021)
	Plant	*Lemna minor*	Triphenylmethane	Crystal violet	0.24					EC_50_ = 0.24 mg/l for chronic 7 days toxicity	*	Adomas et al. (2020)
**Primary consumers**	Ciliate	*Paramecium caudatum*	Xanthene	Rhodamine B	ND	ND	ND	ND	ND	Slow down of movements, accumulation of dye in the contractile vacuole	*	Rowiński and Chrzanowski (2010)
	Cnidaria	*Hydra attenuata*	Triphenylmethane^7^	Auramine					4.8	Alterations in the formation of the tentacles		de Jesus Azevedo et al. (2021)
				Auramine O					1.6			
	Crustacea	*Daphnia magna*	Phenothiazine	Methylene blue		0.149			ND	Affected the heart rate, beat frequency of the thoracic limbs, and reproductive ability	I	Li et al. (2023)
**Primary consumers**	Crustacea	*Daphnia magna*	Triphenylmethane	Malachite green	0.77^4^	ND	ND	ND	ND	Acute immobilisation assay	*	Kanhere et al. (2014)
					ND					Whole growth retardation, inhibited heart rates and cardiac looping		Jang et al. (2009)
			Xanthene	Rhodamine B						Reaction of escape,accumulation of dyes in the intestine, eye; debilitation	*	Rowiński and Chrzanowski (2010)
		*Daphnia similis*	Phenothiazine	Methylene blue	0.16–0.43^3^					ND	I	Fungaroa et al. (2010)
			Triphenylmethane^7^	Auramine	2.9^3^						II	de Jesus Azevedo et al. (2021)
				Auramine O	4.3^3^							
		*Thamnocephalus platyurus*	Xanthene	Rhodamine B	ND	8.06				Strong dyeing, especially of the eye; death	*	Rowiński and Chrzanowski (2010)
	Fish	C*atla catla*^*a*^	Acridine	Acriflavine		60^8^/55^9^	50^8^/47.5^9^	ND	ND	ND	*	Ghosh and Pal (1969)
		*Cirrhinus mrigala*^*a*^				65^8^/60^9^	55^8^/52.5^9^					
		*Danio rerio*				ND	ND			LC_50_ (144 h) = 0.229 mg/L^10^		Meinelt et al. (2002)
		*Danio rerio*^*b*^	Triphenylmethane	Basic fuchsin			0.06			ND		Shen et al. (2015)
		*Danio rerio*^*b*^	Triphenylmethane^7^	Auramine		ND	ND		1.9	Oedema and tail malformations	II	de Jesus Azevedo et al. (2021)
				Auramine O					2.4			
		*Labeo rohita*^*a*^ ^*c*^	Acridine	Acriflavine		80^8^/70^9^	70^8^/65^9^		ND	ND	*	Ghosh and Pal (1969)
			Triphenylmethane	Methyl violet 2B		ND	ND		0.45	Abnormal behaviour, oikilocytosis, altered filaments, lamellae, and rakers of the gill; necrosis	I	Kaur et al. (2016)
		*Oryzias latipes*^*c*^	Phenothiazine	Methylene blue		18	13		ND	ND	*	Tonogai et al. (1982)
			Triphenylmethane	Crystal violet		0.2	0.1					
				Fuchsin		6.8	4.3					
				Malachite green		0.6	0.3					
			Xanthene	Rhodamine B		17	12					
		*Oreochromis niloticus*^*d*^ *Oreochromis niloticus*^*c*^ ^*d*^ ^*x*^ ^*c*^	Acridine	Acriflavine		72.5	58.7	44.1	35.2	Sluggish movement, respiratory distress, restless behaviour: accumulation of fish at water inlet, rapid opercular movement, surfacing, and gasping of atmospheric air	II	Abou-Okada et al. (2021)
**Primary consumers**	Fish		Phenothiazine	Methylene blue	ND	ND	ND	ND	187	Erratic swimming, loss of reflex, restlessness and air gulping	**	Olufayo and Yusuf (2016)
			Triphenylmethane	Malachite green					0.76	Lesions in heptopancreas, kidneys, spleen	I	El-Neweshy and Abou Srag (2011)
								0.45	0.425	ND		Limsuwan (1987)
								ND	0.44	Decreased opercular ventilation, respiratory distress, erratic swimming, death		Omoregie et al. (1998)
**Secondary consumers**	Fish	*Clarias gariepinus*^*a*^	Acridine	Acriflavine	ND	10	ND	ND	ND	ND	II	Obiekezie and Okafor (1995)
	Arthropoda	*Pseudocarcinus gigas*^*e*^	Triphenylmethane	Malachite green		ND				Chronic toxicity < 0.1 mg/l	*	Gardner and Northam (1997)
	Fish	*Anguilla rostrata*^*d*^	Triphenylmethane	Malachite green					0.54	ND	I	Hinton and Eversole (1979)
		*Clarias gariepinus*^*a*^				0.142	ND	ND	ND	ND	I	Obiekezie and Okafor (1995)
		*Cyprinus carpio*^*c*^ *Cyprinus carpio*^*x*^ ^*a*^				0.0265		0.016	0.013			Srivastava et al. (2004a)
						ND	ND	ND	0.135	ND		Limsuwan (1987)
							0.84		ND		*	Svobodova et al. (1997)
		*Heteropneustes fossilis*^*d*^				1	ND	ND	ND	Hyperactivity: rapid pectoral and opercular movement, erratic swimming, gradual loss of equilibrium,breathing difficulties		Srivastava et al. (1995)
						ND			0.24	Hyperactivity, respiratory failure, irregular swimming, refusal to eat	I	Srivastav and Roy (2018)
		*Lepomis macrochirus*^*x*^	Xanthene	Rhodamine B		379			ND	Not observed	*	Marking (1969)
		*Oncorhynchus mykiss*^*d*^				217						
			Triphenylmethane	Malachite green		0.332	0.326	0.306	0.267	Hyperactivity, irregular swimming	I	Van Heerden et al. (1995)
**Tertiary consumers**	Fish	*Ictalurus punctatus*^*x*^	Xanthene	Rhodamine B		526	ND	ND	ND	Not observed	*	Marking (1969)
		*Ophiocephalus striatus*^*x*^	triphenylmethane	Malachite green		0.173			0.069	ND	I	Limsuwan (1987)

^1^EC_50_ is the concentration of a test substance that gives half-maximal response; LC_50_ is the concentration of a test substance that cause the death of 50% of the test organisms; ^2^EC_50_ obtained in 15 min; ^3^EC_50_ obtained in 48 h; ^4^EC_50_ obtained in 24 h; ^5^EC_50_ obtained in 72 h; ^6^*ND*, no data; ^7^auramine and auramine O are diphenylmethane dyes are usually grouped with the triphenylmethane dyes; ^8^LC_50_ obtained in water of temperature 26 °C; ^9^LC_50_ obtained in water of temperature 32 °C, ^10^LC_50_ in conditions: for ↑Ca^2++^, humic substances (-), *lack of data or time of experiment was other than indicated in OECD 2001 to assess toxicity category of test substance. **EC50/LC50 > 100 mg/l. Fish development stages: ^a^fry, ^b^embryo, ^c^fingerling, ^d^juvenile, ^x^age of fish development was not described; ^e^larvae

Among primary consumers, PADs were very toxic to daphnia, with the median lethal concentration (LC_50_) = 0.149 mg/l for methylene blue. Toxicological effects on daphnia include dysfunctions of heart rates, beat frequency of the thoracic limbs, and reproductive ability (Li et al. 2023). As a result, the occurrence of PADs in water bodies disturbs the aquatic food web because the toxicosis of crustacea reduces food resources for higher-level consumers such as crabs, fish, and invertebrate predators (Fig. 3, Table 6) (de Jesus Azevedo et al. 2021).

Acute toxicity of acriflavine, auramine, auramine O, basic fuchsin, crystal violet, fuchsin, malachite green, methyl violet 2B, methylene blue, and rhodamine B in different fish species expressed as LC_50_ is presented in Table 6. The lowest values (< 1 mg/l) of LC_50_ (24 h, 48 h, 72 h, 96 h) were obtained for triphenylmethane dyes: crystal violet (0.1–0.2 mg/l) (Tonogai et al. 1982), methyl violet 2B (0.45 mg/l) (Kaur et al. 2016), and malachite green (0.013–0.84 mg/l) (Limsuwan 1987; Srivastava et al. 2004a) in comparison to acridine dye: acriflavine (35.2–80 mg/l) (Abou-Okada et al. 2021; Ghosh And Pal 1969). 

The majority of toxicity results consider the impact of malachite green on various fish species *Anguilla rostrata* (Hinton And Eversole 1979), *Cyprinus carpio* (Limsuwan 1987; Srivastava et al. 2004a; Svobodova et al. 1997), *Heteropneustes fossilis* (Srivastav And Roy 2018; Srivastava et al. 1995), *Ophiocephalus striatus *(Limsuwan 1987),* Oreochromis niloticus* (El-Neweshy And Abou Srag 2011; Omoregie et al. 1998), and *Oryzias latipes* (Tonogai et al. 1982). The most significant differences in acute toxicity assessment can be observed for different age groups within a given fish species. For young carp (21 days), LC_50_ (96 h) was 0.013 mg/l (Srivastava et al. 2004a), while for adults (> 18 months), LC_50_ (96 h) was 0.135 mg/l (Limsuwan 1987) which shows that young fish are more sensitive to the toxic effects of malachite green. Considering only adults, it can be stated that the species most sensitive to the effects of malachite green is the *Ophiocephalus striatus* (LC_50_ (96 h) = 0.069 mg/l), and among fish breed for consumption purposes—*Cyprinus carpio* (LC_50_ (96 h) = 0.135 mg/l) (Limsuwan 1987) (Table 6).

The toxicity of different PADs, such as acriflavine, methylene blue, and malachite green, was tested on juveniles of Nile tilapia fish (*Oreochromis niloticus*). The obtained results showed that the most toxic was malachite green (0.76 mg/l) (El-Neweshy And Abou Srag 2011) followed by acriflavine (35.2 mg/l) (Abou-Okada et al. 2021) and methylene blue (187 mg/l) (Olufayo And Yusuf 2016). 

Analysis of toxicity data from each level of the consumers (primary, secondary, tertiary), showed that among tested PADs, the most toxic for any level was malachite green (LC_50_ = 0.013–0.6 mg/l) (Table 6). Also, other examples of triphenylmethane dyes, such as basic fuchsin (0.06 mg/l), crystal violet (0.1–1 mg/l), and methyl violet 2B (0.45 mg/l), had toxic potential to aquatic organisms (fish) with LC_50_ ≤ 1 mg/l. On the other hand, the exposition of *Lemna minor* to crystal violet indicated that leaves absorbed dye, preventing light from undergoing photosynthesis. The occurrence of crystal violet caused growth inhibition and changes in the biosynthesis of chlorophyll and resulted in an imbalance of the 1 st trophic level of the aquatic food web (Adomas et al. 2020). These results strongly confirm the toxicological potential of triphenylmethane dyes, particularly malachite green, as a severe water contaminant compared to PADs of other chemical classes.

It should be noted that regardless of the species of fish, the acute toxicological effects of malachite green were similar, including hyperactivity, respiratory failure, irregular swimming, and increased body movement (Omoregie et al. 1998; Srivastav And Roy 2018; Srivastava et al. 2004a; Van Heerden et al. 1995). On the other hand, after applying rhodamine B (xanthene dye), no toxicological effects were observed for fish as secondary and tertiary consumers with LC_50_ = 217 to 526 mg/l. Therefore, rhodamine B dye was the least toxic for tested fish (Marking 1969). However, invertebrates exposure to this dye caused their debilitation and finally death (Rowiński And Chrzanowski 2010) (Table 6).

To predict environmental risk related to dye occurrence in the water, a risk quotient (RQ) method was developed. RQ is a ratio of the measured environmental concentration (MEC) of a chemical substance in the environment to the predicted no effect concentration (PNEC) of the chemical substance below which no harmful effects to the environment are observed. The PNEC is based on EC_50_ or LC_50_ values obtained from acute toxicity data (European Commission 2003). Tkaczyk-Wlizlo and Mitrowska (2023) calculated RQ for crystal violet (0.0122–0.0209 µg/l), methyl violet 2B (0.0274–0.0571 µg/l) found in lake water, and for rhodamine B (0.0594 µg/l) determined in dam water. The obtained values indicated that rhodamine B cannot be classified as a risk, but methyl violet 2B in concentration 0.0571 µg/l and crystal violet in concentration 0.0209 µg/l pose low and medium risk for fish, respectively (Tkaczyk-Wlizlo And Mitrowska 2023). Other available results of environmental risk of PADs are PNEC values obtained for diphenylmethane dyes, auramine (0.92 µg/L) and auramine O (4.6 µg/L) (de Jesus Azevedo et al. 2021). However, the lack of data on MEC did not allow for the calculation of RQ.

## Conclusions

The universality of PADs applications in different areas caused their confirmed occurrence in various types of water and wastewater. Their removal is difficult because the dyes have been designed to resist factors such as light and microbial agents. After treatment, the final effluent of WWTP is directly discharged to the local water environment. Therefore, dye-containing wastewater poses an environmental risk if not treated appropriately.

The awareness of PADs presence in wastewater and their complex degradation in WWTPs caused different methods to be developed to treat them effectively. Still, various approaches have been tested, but there are no guidelines on how to treat it because the actual scale of PADs occurrence in wastewater is unknown and not regulated by law.

The available toxicity studies on aquatic plants and animals have shown that PADs are harmful factors that negatively impact organisms’ development and growth. Moreover, the impact of PADs on the aquatic food web, considering producers and consumers from different levels, has shown dysfunctions in the food chain resulting in trophic imbalance. Thus, PADs in water, especially triphenylmethane dyes with special regard to malachite green, pose a serious environmental problem for aquatic organisms from different levels of a food web. Also, applying dye-containing wastewater or sewage sludge to agriculture is an emerging issue because these dyes permeate to edible parts of plants. Thus, PADs presence in the environment is underestimated and should be monitored to protect humans as top-level consumers of the food web. Therefore, there is a need to establish environmental legislation or norms to control the occurrence of PADs in the aquatic environment and to provide safety.

## Data Availability

Data generated or analysed during this work are provided in full within the published article.
